# Predictive impact of clinical factors on chemosensitivity in advanced high-grade serous ovarian carcinoma according to chemotherapy response score

**DOI:** 10.1097/MD.0000000000040487

**Published:** 2024-11-22

**Authors:** Mia Park, Won Kyo Shin, Myong Cheol Lim, Sang-Yoon Park, Chong Woo Yoo, Kyung-Hee Kim, Kwang-Sun Suh, Heon Jong Yoo

**Affiliations:** a Department of Obstetrics & Gynecology, Chungnam National University Hospital, Daejeon, Republic of Korea; b Department of Obstetrics & Gynecology, Chungnam National University Sejong Hospital, Sejong, Republic of Korea; c Center for Gynecologic Cancer, Research Institute and Hospital, National Cancer Center, Goyang-si, Gyeonggi-do, Republic of Korea; d Department of Pathology, Center for Gynecologic Cancer, Research Institute and Hospital, National Cancer Center, Goyang-si, Gyeonggi-do, Republic of Korea; e Department of Pathology, Chungnam National University School of Medicine, Daejeon, Republic of Korea; f Department of Pathology, Chungnam National University School of Medicine, Chungnam National University Hospital, Daejeon, Republic of Korea; g Department of Obstetrics & Gynecology, Chungnam National University School of Medicine, Chungnam National University Sejong Hospital, Sejong, Republic of Korea.

**Keywords:** chemosensitivity, chemotherapy response score, distant LNs metastases, high-grade serous ovarian carcinoma, neoadjuvant chemotherapy, solid organs metastases

## Abstract

The use of neoadjuvant chemotherapy (NAC) as a first-line therapy for advanced high-grade serous ovarian carcinoma (HGSOC) has increased. However, several studies have reported NAC-induced platinum resistance. This study aimed to evaluate the predictive impact of clinical factors on chemotherapy response score (CRS) and to select patients who would respond well to NAC. This multicenter retrospective (study included patients treated between January 2016 and December 2021). International Federation of Gynecology and Obstetrics stage IIIC and IV HGSOC patients were eligible. Institutionally strict complete resectability criteria were used in the present study. Pathological slides were scored according to the CRS criteria. Among 172 patients with HGSOC, 87 (50.6%) had stage IIIC disease and 85 (49.4%) had stage IV disease. And 35 (20.4%) had CRS1, 103 patients were CRS2 (59.9%), and 34 patients were CRS3 (19.7%). Compared with CRS1, simultaneous metastases to distant lymph nodes and solid organs confirmed by imaging were associated with a 75% reduction in CRS2 (odds ratio = 0.25; 95% confidence interval: 0.09–0.70; *P* = .008). And breast cancer susceptibility gene 1/2 mutation was positively (odds ratio = 8.41; 95% confidence interval: 2.25–31.52; *P* = .002) associated with CRS3 compared to CRS1. Patients with CRS3 had significantly longer progression-free survival (PFS), with median PFS of 9.8, 14.8, and 27.0 months for CRS of 1, 2, and 3, respectively (*P* < .001). Overall survival was also prolonged in patients with CRS3 (*P* < .001). Germline breast cancer susceptibility gene 1/2 mutation was a predictor of CRS3 and a good prognostic factor for the survival rate. Simultaneous metastasis to distant lymph nodes and solid organs is a predictor of CRS1. CRS inversely correlated with PFS and overall survival.

## 1. Introduction

Epithelial ovarian cancer is a lethal type of gynecological cancer, with 313,959 cases diagnosed worldwide every year and 207,252 deaths.^[1]^ Ovarian cancer is associated with a high mortality rate, with a 5-year survival rate of 49% to 51% in Europe and the United States.^[1,2]^ These survival rates drop to 28% to 34% in advanced stages.^[3]^ The most important predictor of survival in advanced-stage disease is the amount of residual tumor after cytoreductive surgery.^[4–7]^ Incomplete resection (residual tumor > 1 cm) has little beneficial effect on survival but is associated with perioperative morbidity. Thus, it is widely agreed that primary surgery in which incomplete resection is expected should be avoided.^[8,9]^ Neoadjuvant chemotherapy (NAC) followed by interval debulking surgery may be a good therapeutic alternative with similar survival rates when primary surgery is deemed not feasible.^[7,8,10,11]^ NAC has gradually increased as first-line therapy, and its use has doubled within 10 years worldwide.^[12–14]^ However, several studies reported cases of NAC-induced platinum resistance and a failure rate of up to 20%.^[15]^ No current validated screening criteria can predict patients who will respond well to NAC before chemotherapy.

Since 2015, the International Collaboration on Cancer Reporting has recommended the use of a standardized histological scoring system for regression.^[16]^ The chemotherapy response score (CRS) for the histological grading of the NAC effect in high-grade serous ovarian carcinoma (HGSOC) is a validated three-tier score that stratifies patients into complete/near-complete (CRS3), partial (CRS2), and no/minimal (CRS1) categories.^[16,17]^ Some studies were published that patients in the CRS3 category showed a statistically significant improvement in progression-free survival (PFS) and overall survival (OS) compared to those in the CRS 1/2 category.^[18–20]^ However, there were few studies on factors that could predict CRS. This study aimed to evaluate the predictive impact of clinical factors on CRS in patients with advanced HGSOC and to select patients who will respond effectively to NAC before treatment.

## 2. Materials and methods

This multicenter, retrospective study included patients diagnosed with HGSOC at Chungnam National University Hospital, Chungnam National University Sejong Hospital, and National Cancer Center in South Korea between January 2016 and December 2021 and was approved by the Institutional Review Board of Chungnam National University Hospital (IRB no. CNUH 2021-06-010) and the Institutional Review Board of the National Cancer Center in Korea (IRB no. NCC 2018-0080). The International Federation of Gynecology and Obstetrics (FIGO) stage IIIC and IV patients with histopathologically confirmed HGSOC were treated with NAC and interval debulking surgery (IDS). Patients were selected according to the strict incomplete resectability criteria of the institution: (i) non-resectable parenchymal liver metastasis; (ii) metastasis to the lungs or mediastinum; (iii) suspicious mesenteric miliary seeding; (iv) metastasis to an unresectable extra-abdominal lymph node (LN); and (v) higher perioperative morbidity or mortality (poor performance status and/or multiple comorbidities). Clinical data included patient age at diagnosis, body mass index (BMI), Eastern Cooperative Oncology Group (ECOG) performance status, cycles of NAC, relative dose intensity, pretreatment serum cancer antigen-125 (CA-125) level, CA-125 reduction level after NAC, germline breast cancer susceptibility gene (BRCA) mutation status, outcome of debulking surgery for the completeness of IDS (surgeon’s visual assessment of completeness of the IDS categorized as no macroscopic residual disease (R0, ≤ 1 cm, and > 1 cm), recurrence, death, and distant metastasis site evaluation using magnetic resonance imaging, computed tomography, and positron emission tomography. Distant metastases that led to the judgment of incomplete resection were classified as distant LN metastases (supraclavicular LN, internal mammary LN, and cardiophrenic LN), solid organ metastases (liver, lung, pancreatic head, and unresectable peritoneal seeding), and simultaneous distant LN and solid organ metastases. NAC was routinely administered as an initial combination of intravenous carboplatin (AUC 5) and paclitaxel (q3 weekly, 175 mg/m^2^) for a mean of 3 cycles. All patients received a standard full dose (relative dose intensity of 100%) of NAC. IDS was performed by midline laparotomy in all cases, and the patients underwent comprehensive surgical staging consisting of a visual inspection of all peritoneal surfaces and prescribed biopsies. All surgeries were performed by gynecologic oncologists and the tumors were sent to highly skilled gynecologic pathologists (KS Suh, KH Kim, and CW Yoo).

Pathology slides from formalin-fixed paraffin-embedded tissue blocks were centrally reviewed by gynecological pathologists, and omental samples with the least NAC response were selected and scored according to CRS criteria for this study^[16]^ where a score of 1 indicated that >95% of the tumors were viable and a score of 3 indicated that <5% were viable. In a very small number of cases, in which it was difficult to distinguish between CRS1 and CRS2 or between CRS2 and CRS3, a consensus score was reached after review and discussion between experienced pathologists. Statistical analysis was performed using the SPSS Statistics 26 software (SPSS Inc., Armonk, NY). Statistical significance was defined as a *P*-value of <.05. The distribution of patient characteristics among the 3 CRS groups was analyzed using the chi-square test (or Fisher’s exact test when the expected frequency within any cell was <5) for categorical variables. The Kruskal–Wallis test was used for continuous variables. Multivariable logistic regression was performed to investigate the prognostic significance of CRS according to age at diagnosis, BMI, ECOG performance status, FIGO stage, pretreatment CA-125 level, BRCA 1/2 mutation status, solid organ metastases, and simultaneous metastases to distant LNs and solid organs. Kaplan–Meier analysis was used to estimate the PFS and OS curves. PFS was defined as the time between the first NAC and the first observation of disease recurrence or death from any cause or for patients who did not have clinical progression within the study census date at the last follow-up consultation. OS was defined as the time from the first NAC administration to death from any cause. The log-rank test was used to assess the significant differences in PFS and OS.

## 3. Results

**Figure 1.**
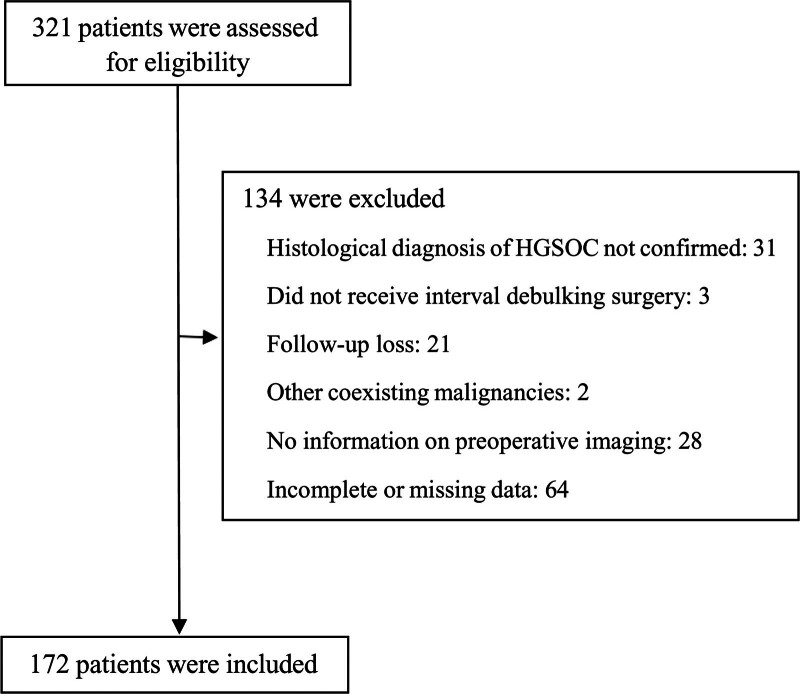
Flow diagram of study participant selection.

We found 321 patients with stage IIIC and IV ovarian cancer who underwent platinum/taxane-based NAC followed by IDS in a multicenter database. One hundred thirty-four patients were excluded: 31 (9.7%) patients had other histopathological diagnoses, 3 patients (0.9%) who received NAC did not undergo subsequent IDS, 21 (6.5%) were lost to follow-up, 2 patients (0.6%) were diagnosed with other coexisting malignancies, 28 (8.7%) had unconfirmed information from preoperative imaging, and 64 (19.9%) had incomplete or missing data (Fig. 1). The remaining 172 patients (53.6%) were included in the study.

Among 172 HGSOC patients treated with NAC and IDS, 87 (50.6%) had stage IIIC disease and 85 (49.4%) had stage IV disease. The demographic characteristics, age at diagnosis, BMI, ECOG performance status, histological subtype, FIGO stage, cycles of NAC, median relative dose intensity, pretreatment CA-125 level, CA-125 reduction level after NAC, BRCA 1/2 mutation status, outcome of debulking surgery (residual disease), and recurrence are shown in Table 1. The distribution of age at diagnosis, BMI, ECOG performance status, histological subtype, FIGO stage, cycles of NAC, and median relative dose intensity did not differ between the groups according to CRS. Pretreatment CA-125 levels were significantly different (2574.7 ± 1647.2, 2775.0 ± 2283.5, and 1739.4 ± 1260.6 U/mL for CRS1, 2, and 3, respectively [*P* = .035]). There were no statistically significant differences between the 3 groups in the absolute levels of CA-125 reduction after NAC (*P* = .168) or in the percentage reduction compared to pretreatment CA-125 levels (*P* = .973). The number of patients diagnosed with the BRCA 1/2 mutation was significantly different among the 3 groups (11.4%, 27.2%, and 50.0% for CRS1, 2, and 3 [*P* = .002]). Most women in this study underwent either complete or optimal cytoreduction, with no gross residual disease (96.5%). There was a statistically significant difference in recurrence among the 3 groups (*P* < .001).

**Table 1:** Baseline patient characteristics according to chemotherapy response score.

	CRS1 (n = 35)	CRS2 (n = 103)	CRS3 (n = 34)	*P*-value*
Age at diagnosis (years)				
Median (range)	61.0 (45.0–79.0)	60.0 (36.0–85.0)	62.0 (41.0–82.0)	.169
BMI (kg/m^2^)				
Mean (SD)	22.3 (3.1)	22.8 (3.6)	23.0 (3.9)	.646
ECOG performance status, n (%)				
0	29 (82.9)	92 (89.3)	30 (88.2)	.612
≥1	6 (17.1)	11 (10.7)	4 (11.8)	
Histology subtype, n (%)				
Papillary serous	35 (100.0)	101 (98.1)	34 (100.0)	.357
Non-papillary serous	0 (0.0)	2 (1.9)	0 (0.0)	
FIGO stage, n (%)				
IIIC	19 (54.3)	52 (50.5)	16 (47.1)	.835
IV	16 (45.7)	51 (49.5)	18 (52.9)	
Cycles of neoadjuvant chemotherapy				
Mean (SD)	3.00 (0.42)	3.09 (0.58)	3.24 (0.85)	.276
Median relative dose intensity (%)	100	100	100	NA
Pretreatment CA-125 (U/mL)				
Mean (SD)	2574.7 (1647.2)	2775.0 (2283.5)	1739.4 (1260.6)	.035
CA-125 reduction level after NAC (U/mL)				
Mean (SD)	2083.7 (1731.6)	2536.9 (2216.8)	1528.8 (976.5)	.168
% (SD)	91.2 (7.5)	91.8 (13.6)	92.4 (12.8)	.973
BRCA 1/2 mutation status, n (%)				
Non-BRCA mutation	31 (88.6)	75 (72.8)	17 (50.0)	.002
BRCA mutation	4 (11.4)	28 (27.2)	17 (50.0)	
Outcome of debulking surgery-residual disease, n (%)				
0 cm (R0)	17 (48.6)	51 (49.5)	23 (67.7)	<.001
>0 cm but ≤ 1 cm	15 (42.9)	50 (48.5)	10 (29.4)	
>1 cm	3 (8.6)	2 (1.9)	1 (2.9)	
Recurrence, n (%)	33 (94.3)	95 (92.2)	20 (58.8)	<.001

*Note*: All results are presented as mean and range or standard deviation (SD) for continuous variables and sample size and percentage for categorical variables.

BMI = body mass index, BRCA = breast cancer susceptibility gene, CA-125 = cancer antigen-125, CRS = chemotherapy response score, ECOG = Eastern Cooperative Oncology Group, FIGO = The International Federation of Gynecology and Obstetrics, NA = not applicable.

* *P*-values were determined by one-way analysis of covariance for continuous variables and chi-square test or fisher’s exact test for categorical variables.

**Figure 2.**
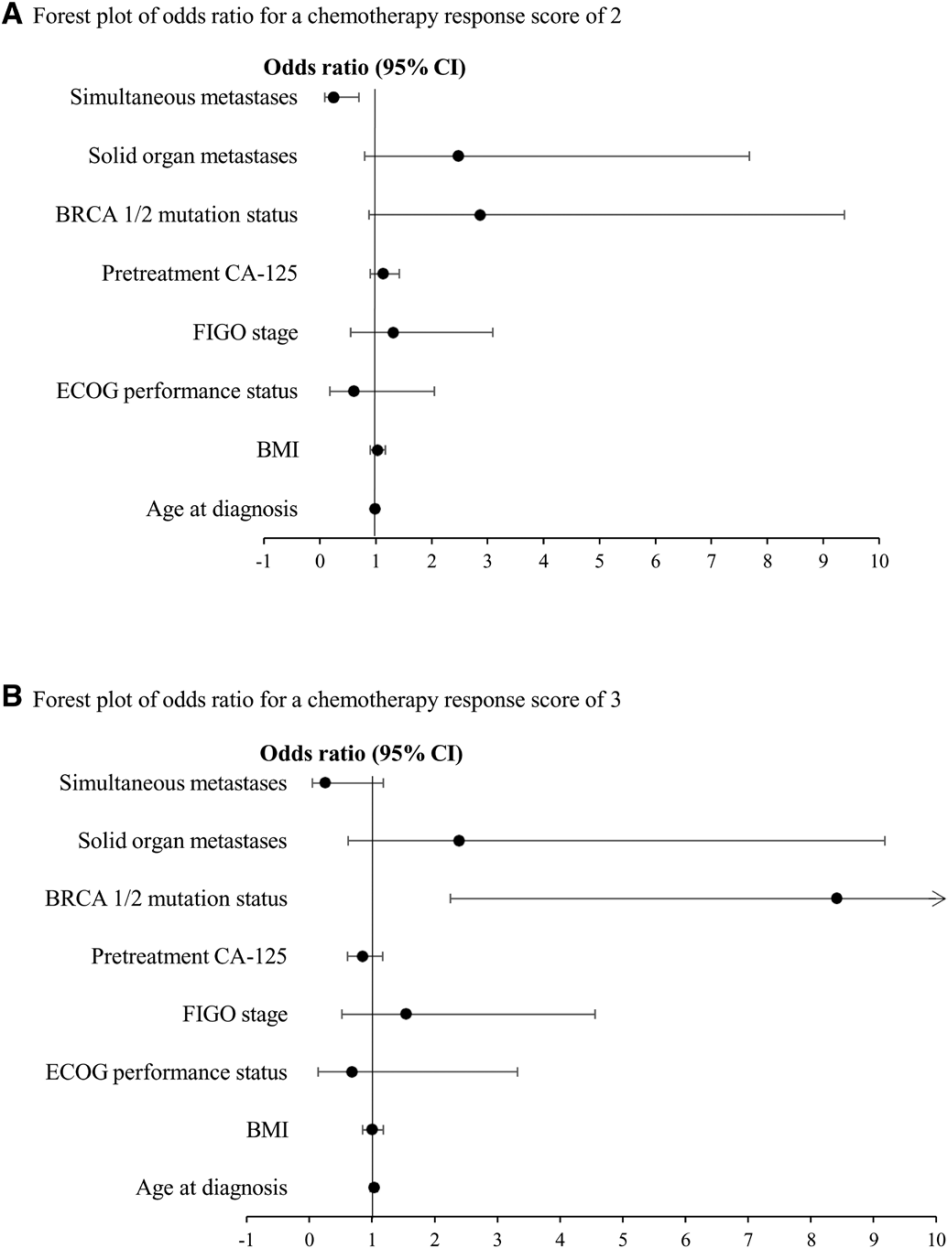
Forest plot of odds ratio for (A) chemotherapy response score of 2 and chemotherapy response score of 3.

Univariate and multivariate multiple logistic regression analyses were conducted to identify predictive factors for chemosensitivity according to the CRSs. These variables included age at diagnosis, BMI, ECOG performance status, FIGO stage, pretreatment CA-125, BRCA 1/2 mutation status, solid organ metastases, simultaneous metastases to distant LN, and solid organ metastases confirmed by imaging (Table 2). Multivariable multiple logistic regression analysis showed that simultaneous metastases to distant LNs and solid organs confirmed by imaging had an inversely significant effect on CRS2 (odds ratio [OR] = 0.25; 95% confidence interval [CI]: 0.09–0.70; *P* = .008) compared to CRS1. In addition, the BRCA 1/2 mutation was positively (OR = 8.41; 95% CI: 2.25–31.52; *P* = .002) associated with CRS3 compared to CRS1 (Fig. 2).

**Table 2:** Univariable and multivariable logistic regression analysis of predictive factors for chemotherapy response scores (CRS) 2 and 3 compared to CRS1.

Predictor	Univariable analysis				Multivariable analysis			
	CRS2	*P*-value	CRS3	*P*-value	CRS2	*P*-value	CRS3	*P*-value
	OR (95% CI)		OR (95% CI)		OR (95% CI)		OR (95% CI)	
Age at diagnosis	0.99 (0.95–1.03)	.612	1.03 (0.98–1.08)	.251	0.99 (0.95–1.04)	.782	1.03 (0.97–1.09)	.310
BMI	1.05 (0.94–1.17)	.418	1.06 (0.93–1.22)	.378	1.03 (0.90–1.17)	.698	1.00 (0.85–1.18)	.979
ECOG performance status	0.58 (0.20–1.70)	.319	0.64 (0.17–2.52)	.528	0.61 (0.18–2.05)	.423	0.68 (0.14–3.32)	.637
FIGO stage	1.17 (0.54–2.51)	.698	1.34 (0.52–3.44)	.549	1.31 (0.55–3.09)	.542	1.54 (0.52–4.56)	.433
Pretreatment CA-125	1.05 (0.87–1.27)	.625	0.76 (0.57–1.01)	.058	1.13 (0.90–1.42)	.302	0.85 (0.61–1.17)	.306
BRCA 1/2 mutation status	2.89 (0.94–8.94)	.065	7.75 (2.24–26.76)	.001	2.87 (0.88–9.38)	.080	8.41 (2.25–31.52)	.002
Solid organ metastases confirmed by imaging	1.91 (0.68–5.42)	.222	1.92 (0.59–6.31)	.282	2.48 (0.8–7.68)	.116	2.39 (0.62–9.18)	.206
Simultaneous metastases to distant LNs and solid organs confirmed by imaging	0.22 (0.09–0.59)	.002	0.17 (0.04–0.69)	.013	0.25 (0.09–0.70)	.008	0.25 (0.05–1.18)	.079

*Note*: Multinomial logistic regression models were used to estimate odds ratios, corresponding 95% CIs, and *P* values for CRS 2 and 3 versus CRS 1. Pretreatment CA-125 was examined per 1000-unit increase.

BMI = body mass index, BRCA = breast cancer susceptibility gene, CA-125 = cancer antigen-125, CI = confidence interval, CRS = chemotherapy response score, ECOG = Eastern Cooperative Oncology Group, FIGO = The International Federation of Gynecology and Obstetrics, OR = odds ratio.

**Figure 3.**
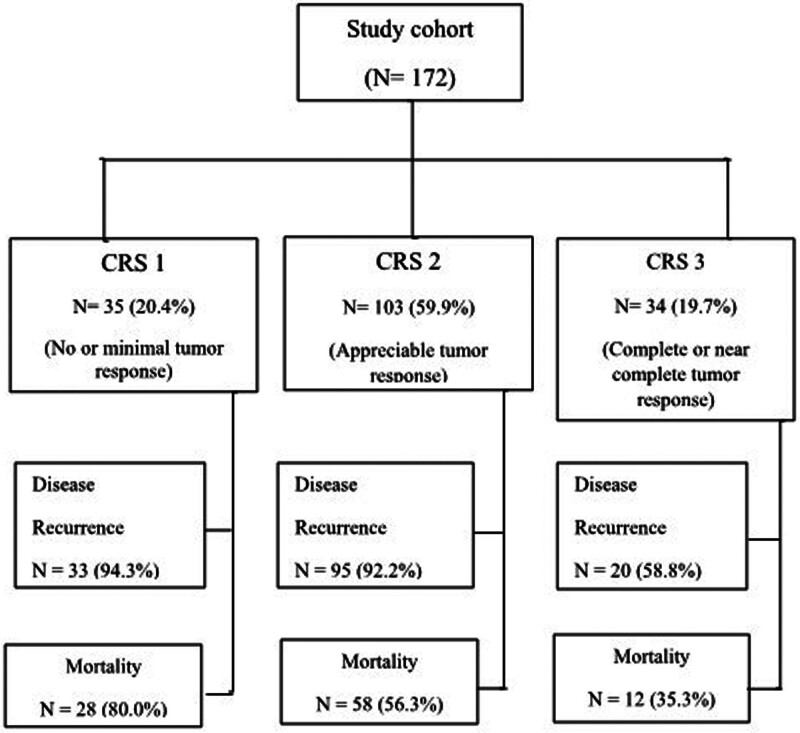
Distribution of chemotherapy response scores (CRS) and recurrence and mortality rates by CRS in the study cohort.

**Figure 4.**
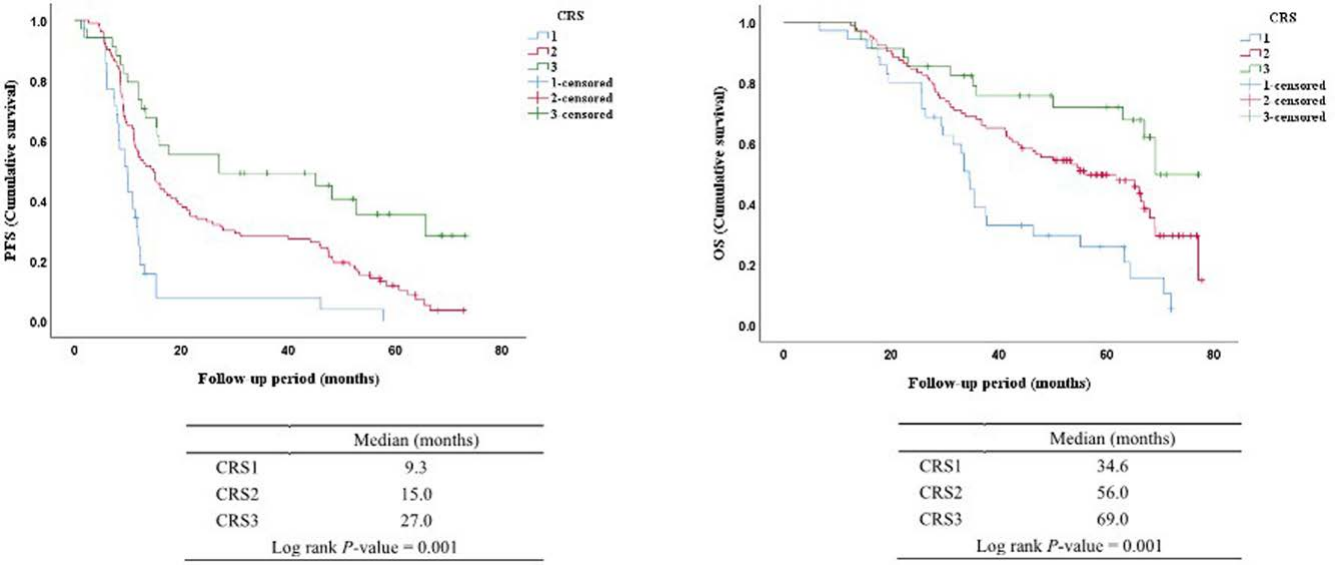
Kaplan–Meier curves for progression-free (PFS) survival outcomes and overall survival (OS) per chemotherapy response score (CRS).

The distribution of CRS in the study cohort and recurrence and mortality rates according to CRS are shown in Figure 3. Among the 172 patients, 35 (20.4%) had CRS1, 103 patients were CRS2 (59.9%), and 34 patients were CRS3 (19.7%). Disease recurrence and mortality were highest in patients with a CRS of 1 (94.3% and 80.0%, respectively) and lowest in those with a CRS of 3 (58.8% and 35.3%, respectively). The median follow-up was 44.8 months (interquartile range, 27.1 to 63.3). The Kaplan–Meier survival curves for PFS and OS per CRS are shown in Figure 4. PFS was significantly longer in patients with CRS3, with median PFS of 9.8, 14.8, and 27.0 months for CRS of 1, 2, and 3, respectively (*P* < .001). OS was also longer for patients with CRS3 (median OS = 34.5, 56.0, and 69.0 months for CRS of 1, 2, and 3, respectively [*P* < .001]).

## 4. Discussion

In the present study, simultaneous metastases to distant LNs and solid organs confirmed by imaging were significantly associated with chemotherapy-resistant CRS1, and BRCA 1/2 mutation were shown to correlate with CRS3 compared to those with CRS1. The PFS and OS were longer in patients with CRS3. Therefore, it is advisable to consider NAC first in patients with predicted high CRS. This is the first study to predict CRS in patients diagnosed with HGSOC solely based on pretreatment clinical factors.

The most common distant metastasis sites of ovarian cancer are the liver, distant LNs, and lungs, with the exception of peritoneal seeding.^[21,22]^ Although few studies have analyzed patient prognosis by the distant metastasis site,^[23]^ there has been no previously published study on the relationship between distant metastasis and chemotherapy response. This study is the first to analyze this relationship. Simultaneous metastases to distant LNs and solid organs were significantly associated with CRS1 (OR = 0.25; 95% CI: 0.09–0.70; *P* = .008). This may be because aggressive cancer cells show a low chemotherapy response and a tendency to metastasize to distant organs and LNs. This finding needs to be confirmed in a larger study population.

Some studies have recommended using CRS in patients with HGSOC to indicate the pathologic response to chemotherapy.^[16–18]^ Several studies have been conducted on the inter-observer reproducibility of CRS read blindly by 2 or more pathologists. The reported reproducibility was high, especially in identifying patients with a CRS of 3 with the best chemotherapy response.^[19,24,25]^ However, other studies reported discrepancies in reproducibility.^[17,26]^ In the present study, a central review was performed by skilled gynecologic pathologists. Most of the results were clear, and a consensus score was reached for a very small number of slides, with conflicting readings through review and discussion. Therefore, the reliability and reproducibility of this study were improved.

Many studies have been conducted to determine the relationship between CRS and survival rate. Recent studies have shown that CRS3 expression is a strong predictor of PFS and OS.^[18,27,28]^ Similarly, in the present study, PFS and OS were inversely correlated with CRS. This study confirmed the prognostic significance of CRS scores in advanced ovarian cancer by identifying higher PFS and OS in patients with CRS3. These results suggest that CRS is a reliable predictor of survival in patients with advanced HGSOC.

Previous studies have investigated the CA-125 reduction rates and patient prognosis after chemotherapy.^[29,30]^ They reported that the higher the CA-125 reduction rate, the better the prognosis.^[29,30]^ In this study, the percentage of CA-125 reduction compared to pretreatment levels was higher in patients with CRS3, but it was not statistically significant (91.2, 91.8, and 92.4% for CRS of 1, 2, and 3, respectively [*P* = .973]). These findings suggest that the pathologic response is more sensitive than the clinical response, or it might be that the pathologic response and the clinical response do not correlate. Further studies are required to confirm these results.

Other studies have reported a relationship between pretreatment CA-125 levels and prognosis. Xu et al reported that patients with high baseline CA-125 levels (424–1484 U/mL) had a high probability of relapse, even if a complete clinical response occurred, and patients should be followed with caution.^[31]^ Markedly elevated CA-125 levels (>500 U/mL) were associated with a low likelihood of achieving optimal cytoreduction, suggesting that elevation in this ovarian cancer biomarker is associated with tumor aggressiveness.^[32–35]^ In contrast, in a Southwest Oncology Group study of 101 patients with advanced ovarian cancer, CA-125 levels before chemotherapy were not associated with survival.^[32,35,36]^ In this study, pretreatment CA-125 levels were statistically significantly different (2574.7 ± 1647.2, 2775.0 ± 2283.5, and 1739.4 ± 1260.6 U/mL for CRS1, 2, and 3, respectively [*P *= .035]). The value of pretreatment CA-125 level as a prognostic marker should be confirmed in a larger population. Our study had certain limitations. First, data were retrospectively collected. The possibility of selection bias and other confounding factors exists in this study. Second, we were unable to collect relevant clinical details, such as the patient’s choice to discontinue the treatment. Despite these limitations, this is the first study to attempt to predict CRS based on clinical characteristics before treatment. In addition, this study revealed an association between BRCA mutations and CRS through a central pathological review. Second, as shown in Table 2, in the univariate analysis, there was a significant inverse association in CRS3 values compared to CRS1 when simultaneous metastases were present (OR = 0.17; 95% CI: 0.04–0.69; *P* = .013), but in multivariate analysis, there was a discrepancy with no association (OR = 0.25; 95% CI: 0.05–1.18; *P* = .079). This result may have been influenced by variable interactions. It is possible that multivariable analysis was insufficient to detect the effect observed in univariate analysis with a small sample size, so there is a need for future studies with a larger sample size. Although this result is insufficient to support the prediction of a complete response to chemotherapy, it is considered to have clinical significance as a result of the prediction of a partial response, as most patients with HGSOC have CRS2.

In conclusion, germline BRCA 1/2 mutation was a predictor of CRS3 and a good prognostic factor for the survival rate. Simultaneous metastasis to distant LNs and solid organs is a predictor of CRS1. This study will be a valuable reference for determining whether to administer NAC to patients with advanced HGSOC in clinical practice.

## Acknowledgments

We appreciate the in-depth review of the statistical and methodological accuracy of this study performed by the Academic Clinical Research Operating and Supporting System of Chungnam National University Hospital Biomedical Research Institute.

## Author contributions

**Conceptualization:** Won Kyo Shin, Heon Jong Yoo.

**Data curation:** Won Kyo Shin.

**Formal analysis:** Mia Park.

**Funding acquisition:** Heon Jong Yoo.

**Investigation:** Mia Park, Won Kyo Shin.

**Methodology:** Won Kyo Shin, Chong Woo Yoo, Kyung-Hee Kim, Kwang-Sun Suh.

**Supervision:** Myong Cheol Lim, Sang-Yoon Park, Kwang-Sun Suh, Heon Jong Yoo.

**Visualization:** Mia Park.

**Writing – original draft:** Mia Park.

**Writing – review & editing:** Myong Cheol Lim, Sang-Yoon Park, Heon Jong Yoo.
